# Supplementary material for: Effect of immune modulation on the skeletal muscle mitochondrial exercise response: An exploratory study in mice with cancer
Source: PLoS One. 2021 Oct 19;16(10):e0258831. doi: 10.1371/journal.pone.0258831 (PMC8525738; doi:10.1371/journal.pone.0258831)
Supplement: S1 Fig — Representative whole blots probed for GAPDH of muscle lysates from tumor-free mice (a) or mice with B16-F10 (b) or EO771 tumors (c), and the densitometric quantification thereof (d-f). n = 8–12 per group. Data are shown as individual data points and mean with 95% CI. Data analysed by unpaired, two-tailed student’s t test (tumor-free and B16-F10) or Mann-Whitney test (EO771). (PDF) [file pone.0258831.s001.pdf]

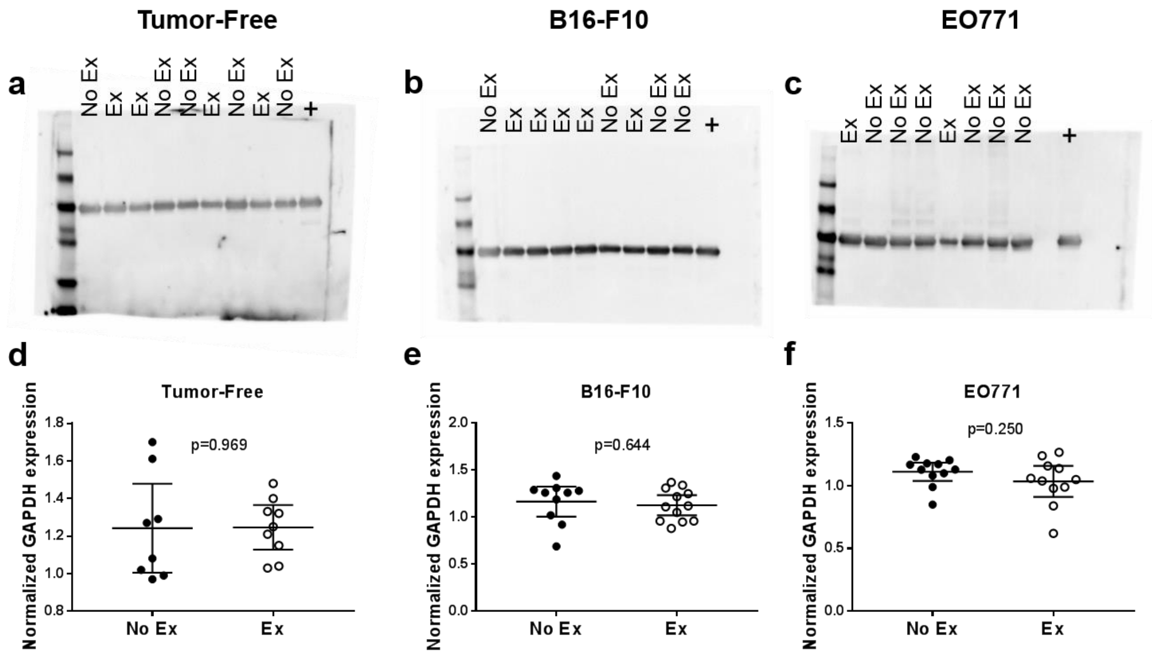

Supplementary Figure 1: Muscular GAPDH protein expression is not altered by exercise.

Representative whole blots probed for GAPDH of muscle lysates from tumor-free mice **(a)** or mice with B16-F10 **(b)** or EO771 tumors **(c)**, and the densitometric quantification thereof **(d-f)**. n=8-12 per group. Data are shown as individual data points and mean with 95% CI. Data analysed by unpaired, two-tailed student's t test (tumor-free and B16-F10) or Mann-Whitney test (EO771).
