# Supplementary material for: Effect of immune modulation on the skeletal muscle mitochondrial exercise response: An exploratory study in mice with cancer
Source: PLoS One. 2021 Oct 19;16(10):e0258831. doi: 10.1371/journal.pone.0258831 (PMC8525738; doi:10.1371/journal.pone.0258831)
Supplement: S1 Raw images — (PDF) [file pone.0258831.s002.pdf]

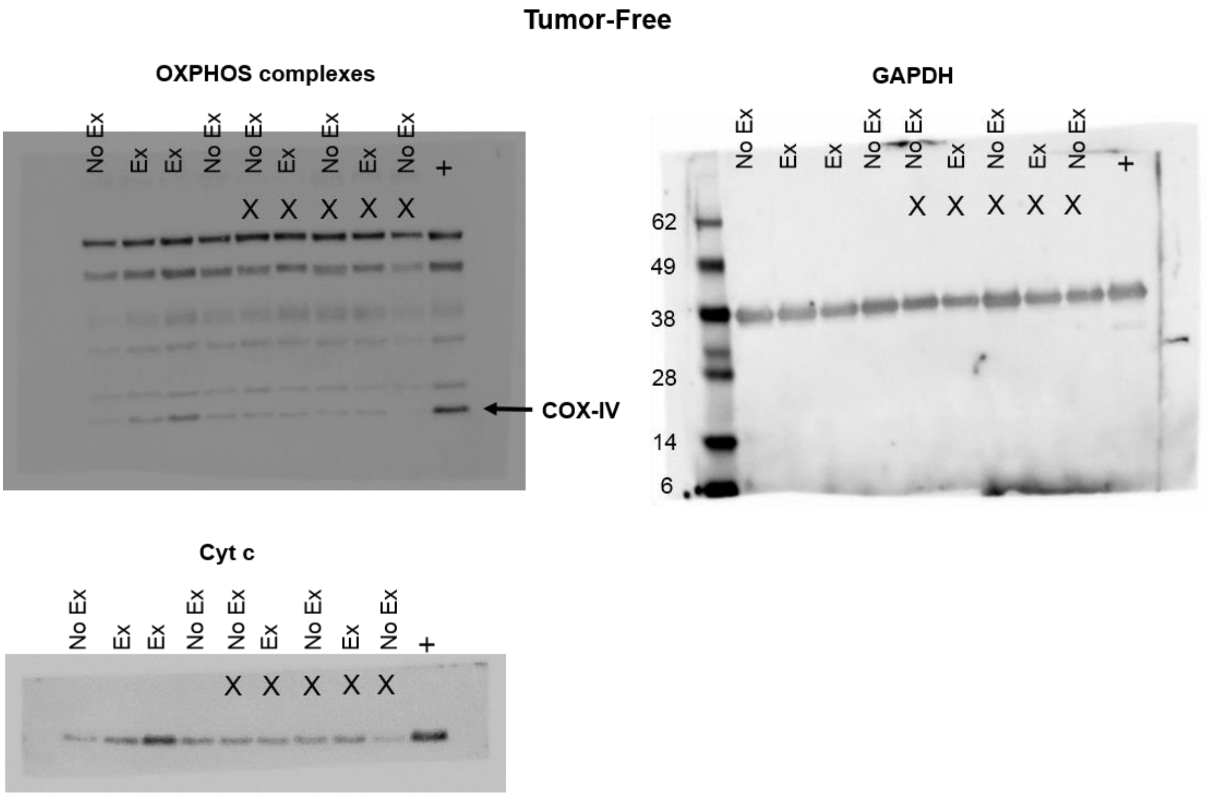

Figure 1: Full blots used in Fig 2.

Images showing OXPHOS/COX-IV and GAPDH are from the same blot. Anti-COX-IV, GAPDH and OXPHOS antibodies added simultaneously. OXPHOS/COX-IV and GAPDH detected separately using secondary antibodies labelled with IRDye 800CW and IRDye 680RD, respectively. Cytochrome c (cyt c) is from a separate blot which was cut between the 14 kDa and 28 kDa band to probe for GAPDH and cyt c separately. X indicates lanes that are not shown in the final figure.

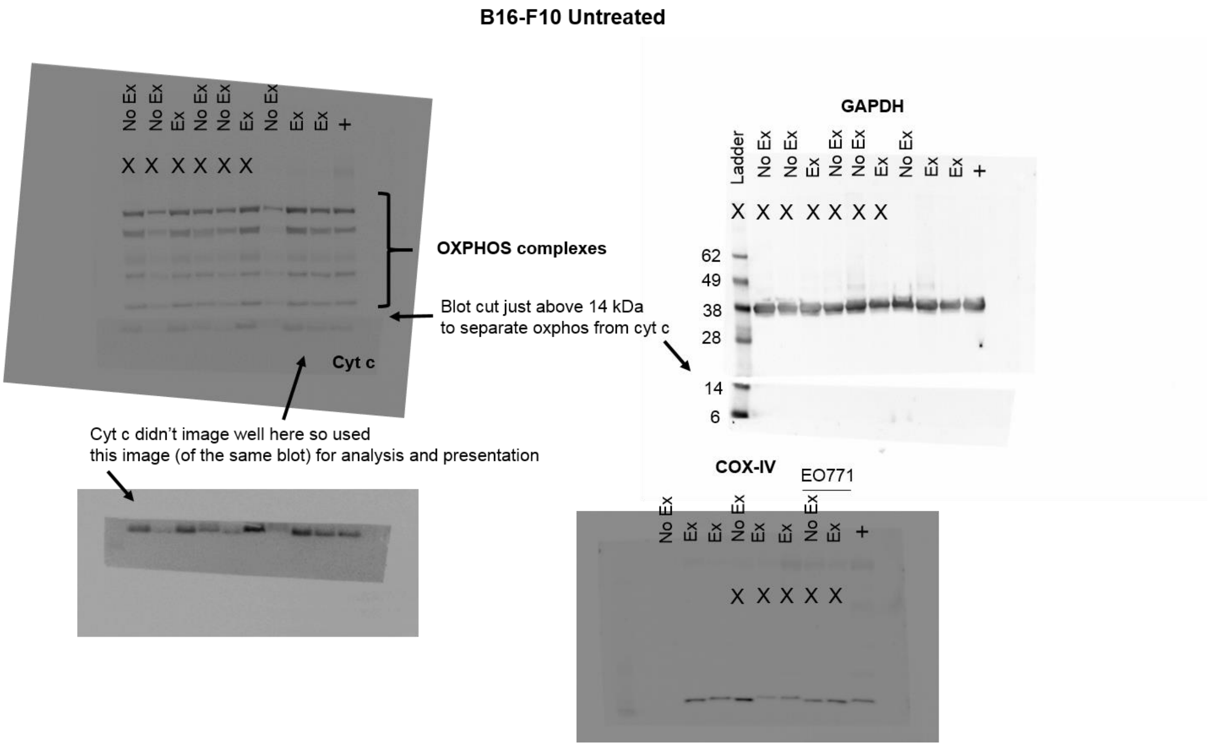

Figure 2: Full blots used in the first panel in Fig 3 (B16-F10 untreated).

Images showing OXPHOS, cyt c and GAPDH are from the same blot. GAPDH and OXPHOS antibodies added simultaneously. OXPHOS and GAPDH detected separately using secondary antibodies labelled with IRDye 800CW and IRDye 680RD, respectively. Image showing COX-IV is from a separate blot. X indicates lanes that are not shown in the final figure.

B16-F10 IgG2a/aPD-1 treated

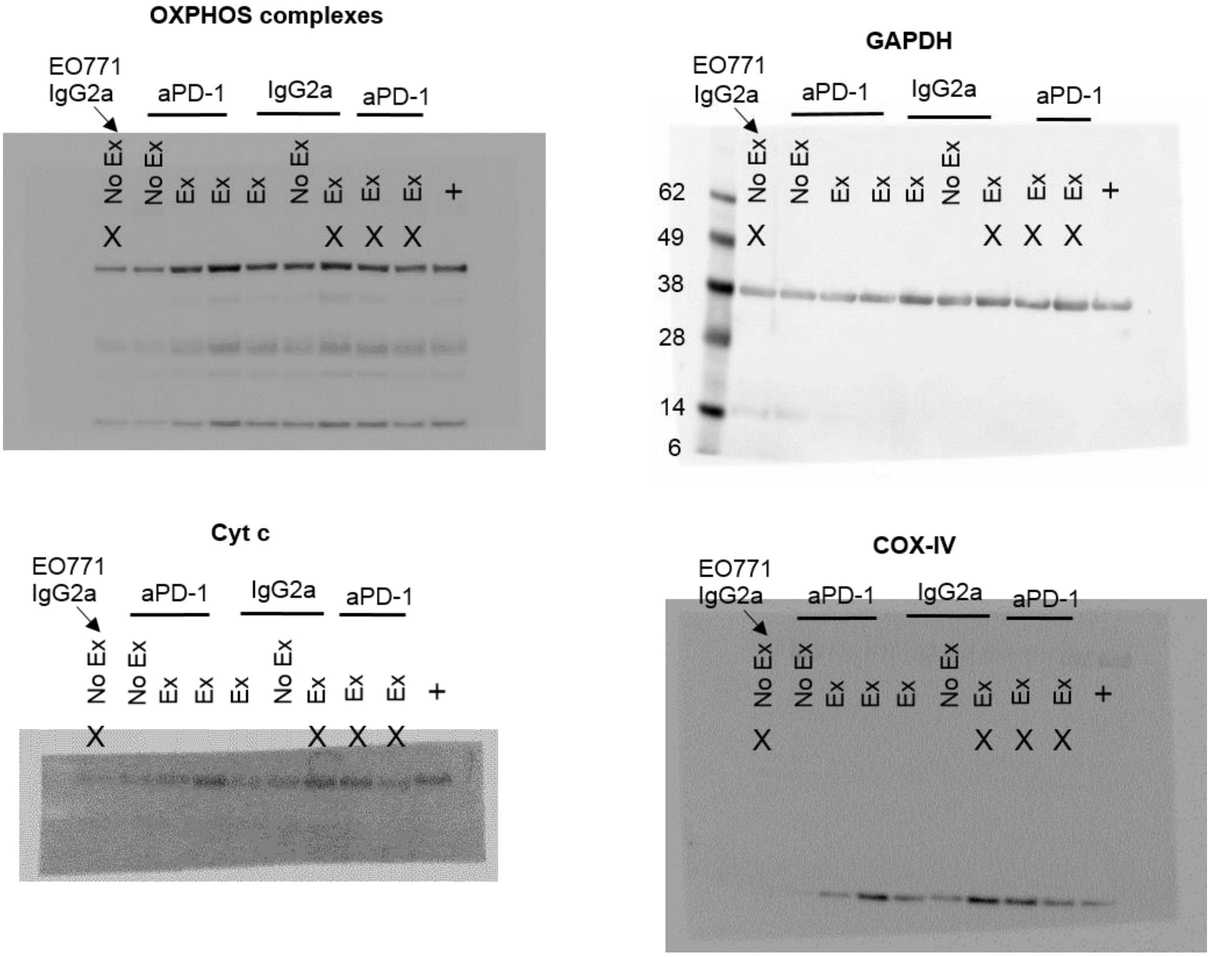

Figure 3: Full blots used in the second panel in Fig 3.

Images showing OXPHOS and cyt c are from the same blot. Blot was cut just above the 14 kDa band to separate cyt c from OXPHOS complexes. Images showing COX-IV and GAPDH are from the same blot. GAPDH and COX-IV antibodies added simultaneously. COX-IV and GAPDH detected separately using secondary antibodies labelled with IRDye 800CW and IRDye 680RD, respectively. X indicates lanes that are not shown in the final figure.

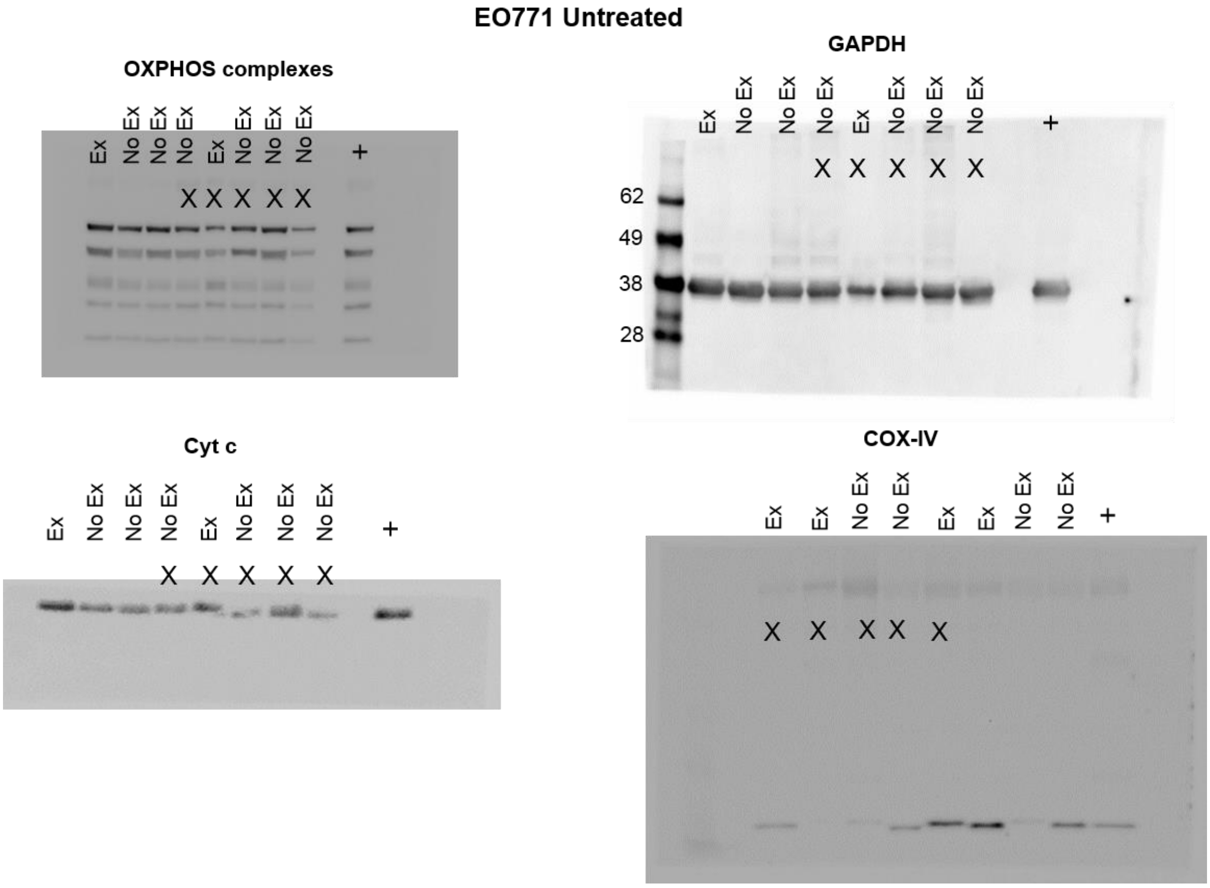

Figure 4: Full blots from the first panel in Fig 4.

Images showing OXPHOS, GAPDH and cyt c are from the same blot. Blot was cut just above the 14 kDa band to separate cyt c from OXPHOS complexes. GAPDH and OXPHOS antibodies added simultaneously. OXPHOS and GAPDH detected separately using secondary antibodies labelled with IRDye 800CW and IRDye 680RD, respectively. Image showing COX-IV is from a separate blot. X indicates lanes that are not shown in the final figure.

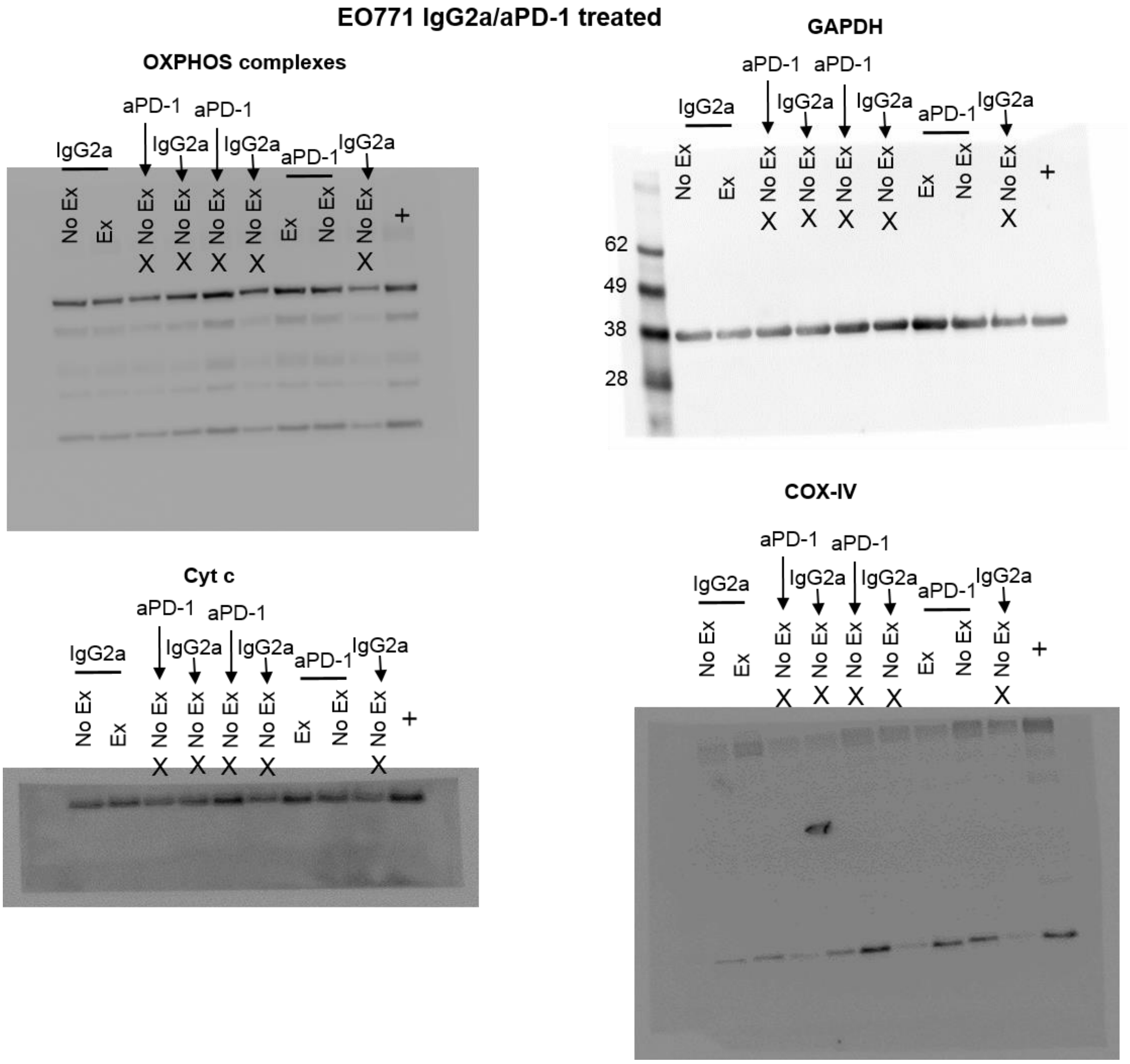

Figure 5: Full blots from second panel in Fig 4.

Images showing OXPHOS, GAPDH and cyt c are from the same blot. Blot was cut just above the 14 kDa band to separate cyt c from OXPHOS complexes. GAPDH and OXPHOS antibodies added simultaneously. OXPHOS and GAPDH detected separately using secondary antibodies labelled with IRDye 800CW and IRDye 680RD, respectively. Image showing COX-IV is from a separate blot. X indicates lanes that are not shown in the final figure.

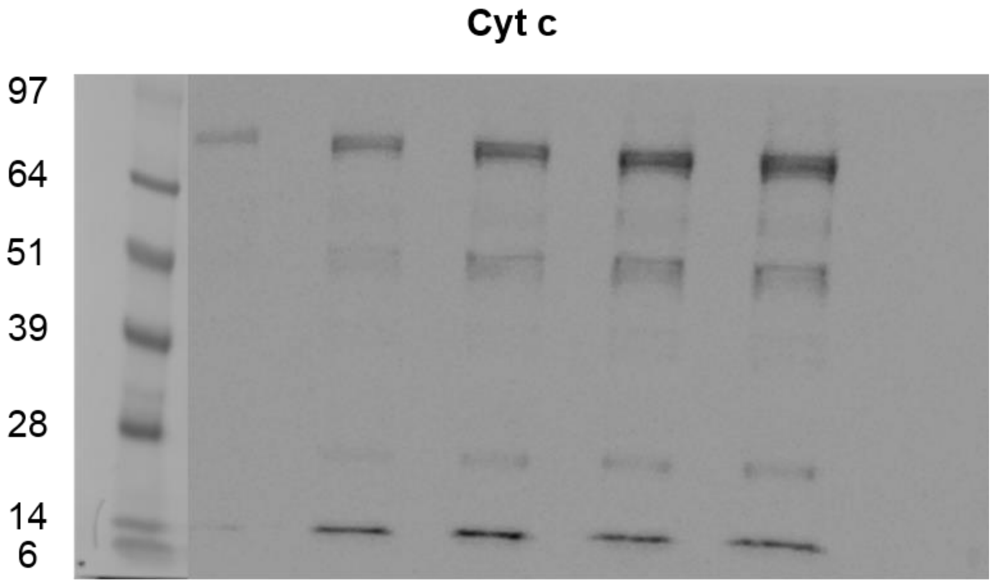

Figure 6: Representative full blot probed for cytochrome c.

Note: molecular weight markers are slightly different than in the final blots as this gel was run using MOPS buffer, while the gels used for analysis were run using MES buffer. This image is only provided as an example of the background bands observed when probing a whole blot for cytochrome c.
