# Supplementary material for: Effect of immune modulation on the skeletal muscle mitochondrial exercise response: An exploratory study in mice with cancer
Source: PLoS One. 2021 Oct 19;16(10):e0258831. doi: 10.1371/journal.pone.0258831 (PMC8525738; doi:10.1371/journal.pone.0258831)
Supplement: Attachment — Submitted filename: Buss_Response to Reviewers final.docx [file pone.0258831.s003.docx]

Dr Atsushi Asakura

Academic Editor

PLOS ONE	5^th^ May 2021

Dear Dr Asakura

*Resubmission of manuscript entitled: ‘Effect of immune modulation on the skeletal muscle mitochondrial exercise response: an exploratory study in mice with cancer’, by Buss et al.*

Thank you for forwarding the reviewer’s comments, and giving us the opportunity to resubmit our manuscript. Below are our detailed responses to the journal requirements and reviewers’ comments (indented in blue).

Journal Requirements:

1. Please ensure that your manuscript meets PLOS ONE's style requirements, including those for file naming.

We have formatted our manuscript to meet the style requirements as requested. If we have inadvertently missed anything, please do not hesitate to let us know.

2. Thank you for stating the following in the Financial Disclosure section:

"We appreciate funding from the Mackenzie Charitable Foundation (MJC, GUD, BAR), the University of Otago (doctoral scholarship for LAB) and Saunders Robinson Brown (McGee Fellowship for LAB). The funders had no role in study design, data collection and analysis, decision to publish, or preparation of the manuscript."

We note that you received funding from a commercial source:  Saunders Robinson Brown.

Please provide an amended Competing Interests Statement that explicitly states this commercial funder, along with any other [relevant declarations](about:blank) relating to employment, consultancy, patents, products in development, marketed products, etc.

This has been a misunderstanding. We did not receive any funding from a commercial source. Saunders Robinson Brown manage the funds in the McGee Fellowship Fund, which was established to support students undertaking postgraduate research into neurological, respiratory or muscle wasting disorders at the University of Otago, Christchurch and functions as a trust. **It would therefore be more accurate to say that we received funding from the McGee Fellowship Fund. We would therefore like to amend our financial disclosure statement to:**

"We appreciate funding from the Mackenzie Charitable Foundation (MJC, GUD, BAR), the University of Otago (doctoral scholarship for LAB) and the McGee Fellowship Fund (LAB). The funders had no role in study design, data collection and analysis, decision to publish, or preparation of the manuscript."

We therefore do not need to amend the Competing Interests Statement.

3. PLOS ONE now requires that authors provide the original uncropped and unadjusted images underlying all blot or gel results reported in a submission’s figures or Supporting Information files. This policy and the journal’s other requirements for blot/gel reporting and figure preparation are described in detail at <https://journals.plos.org/plosone/s/figures#loc-blot-and-gel-reporting-requirements> and <https://journals.plos.org/plosone/s/figures#loc-preparing-figures-from-image-files>. When you submit your revised manuscript, please ensure that your figures adhere fully to these guidelines and provide the original underlying images for all blot or gel data reported in your submission. See the following link for instructions on providing the original image data: <https://journals.plos.org/plosone/s/figures#loc-original-images-for-blots-and-gels>.

In your cover letter, please note whether your blot/gel image data are in Supporting Information or posted at a public data repository, provide the repository URL if relevant, and provide specific details as to which raw blot/gel images, if any, are not available. Email us at [plosone@plos.org](mailto:plosone@plos.org) if you have any questions.

**The original blot images were provided with the original submission in the supporting document S2_raw_images.** We believe that these meet your requirements. If not, we would be happy to amend them if you provide us with additional information as to which requirements are not being met.

Reviewer #1: You showed that tumor burden may dysregulate complex IV assembly and affecting overall function of the OXPHOS chain. Does anti-PD1 or any antibody (IgG2) have any effect (positive or negative) on this?

Thank you for a great question! **We have now performed this additional analysis and have added the results to the manuscript (new Table 5).** It appears that both the isotype control and anti-PD-1 may partially reverse the dysregulation that might be occurring with tumour burden.

Reviewer #2: 
Line 56:
“prior to any noticeable muscle wasting in both mice [2] and humans [3], suggesting that mitochondrial dysfunction plays a role in the pathogenesis of cancer cachexia”

There is evidence in ref 2, for a dysfunction of mitochondrial function before a measurable mass loss. This does not mean that there is a suggestion of a possibly causality between mitochondrial disfunction and muscle loss in cachexia. Others researcher have made an extensive review for the possible pathways that induce mitochondrial dysfunction in cancer cachexia (BBA - Reviews on Cancer 1870 (2018) 137–150). Their revision of multiple available data indicates that cancer is a process able to induce cachexia and mitochondrial dysfunction. Even if this state may constitute a negative loop for muscle loos, I suggest the author of the present manuscript to be careful with sentences that implies causality.

Our aim with this sentence was not to imply that mitochondrial dysfunction causes muscle mass loss, but rather to suggest that this is one of the earliest processes being dysregulated by tumour burden and therefore may be an early indicator, as it is present before the usual clinical indicators such as muscle mass loss.

In response, **we have amended the sentence to read “…suggesting that mitochondrial dysfunction may play a role in the pathogenesis of cancer cachexia and serve as an early indicator of its onset” (lines 65 and 66)**, which we hope is clearer.

Line 91
Due to the role of the immune system in muscle regeneration and adaptation to exercise, it is possible that immunotherapies such as anti-PD-1 will affect muscle responsiveness to exercise training.
A couple of references are needs here.

**We have inserted references as requested.**

Also, it has to be clear if the cellular mechanism of this role of immune system to adaptation of skeletal muscle to exercise have been reported or if the evidence is only circumstantial.

This has been discussed and referenced in the preceding paragraph (lines 102-112). **We have amended the text to clarify this issue (lines 119-120).**

Results:
In table 1, changes in weight of mice are shown. No significant changes were reported between mice before and post exercise for initial weight and final weight. However, authors reported a significant change in % of body weight change. This result is not coherent with the first data. If change of weight is a quotient between initial and final weight, how could appear a significance? An error in statistical analysis appears most likely. If not, please explain.

The difference in statistical significance can be explained by the fact that the change in body weight is a measure for the *difference* between initial and final body weight within a group, which has then been compared between exercising and non-exercising groups, while the comparison between initial and final weights across exercising and non-exercising mice do not incorporate this difference. To further illustrate this, **we have performed a paired t test between initial and final body weight for non-exercising (p<0.0001) and exercising (p=0.30) mice (Table 1 has been updated).** As can be seen, the test shows that the increase in body weight in non-exercising mice is statistically significant, while that in exercising mice is not. This is what is being shown by the statistically significant difference in % change in body weight between non-exercising and exercising mice.

Is important to treat exercise as a multifactorial stimulus, in particular in reference to endurance or strengthen exercise type. In the works used as references, clearly used an endurance type exercise, as the pathways activated correspond to those obtained with tonic electrostimulation or endurance training, exercise that induce plasticity changes from fast type to slow type muscle fibers, which have a higher content in mitochondria a higher level of mitochondrial enzymes related to oxidative metabolism. So, the first experiment that the authors should be do, is a control of the modification of muscle type and/or mitochondria content and function, obtained with the exercise in wheel. Otherwise, we can’t know if the exercise choose is, in control animals, inducing the expected results.

We had performed this experiment, as suggested. This initial experiment is shown in Figure 2, where we have investigated the effect of exercise on the expression of mitochondrial markers and activity of mitochondrial enzymes in tumour-free control mice. This was then used as a benchmark to interpret our results in tumour-bearing mice (see for example lines 341-343, “This suggests that the skeletal muscle mitochondrial exercise response is intact in mice bearing B16-F10 melanoma, as the response was similar to that seen in tumor-free mice”). As this was an exploratory, biochemical analysis, the characterisation of muscle fibre type was beyond the scope of this investigation and we no longer have sufficient samples available for retrospective analysis. However, we agree that this would be a valuable analysis to add to any future investigation.

My main critic to this work is the conceptual design referring to exercise. If the author were searching for an exercise type that increases mitochondrial contain, they should in the first place demonstrate that the exercise chosen induced the change in mitochondrial content and function searched. This is not done.

Please see our response to your previous comment, illustrating that we have, in fact, shown a change in mitochondrial content with our chosen exercise type in control mice (Figure 2). Additionally, it is extensively documented in the literature that endurance exercise, such as voluntary wheel running, induces increases in mitochondrial content. **We have added and referenced lines 314-315 to clarify this in the manuscript**. Therefore, we are confident that our chosen exercise type induced the required changes. We note that our training duration was relatively short, but as we show in Figure 2, it was nevertheless sufficient to induce some increases in mitochondrial content markers.

The markers for mitochondria are poor and the techniques used are weak also. I miss muscle slides to evaluate the histology of the muscle, the infiltration content, fiber integrity, fiber cross sectional area and also other markers as SDH, LDH, and general master genes as PGC1-alpha.

The chosen markers relate to an essential mitochondrial function, namely oxidative phosphorylation, and so reflect changes in mitochondrial health. The techniques (Western blot and enzyme assays) are well-established methods commonly used in the field. **We have clarified this in the manuscript, lines 177-179 and 205-206.**

We accept that Western blotting is semi-quantitative, but we have used the same positive control sample on all blots, as well as a loading control, to make the results as accurate and comparable to each other as possible.

Now that we have shown, for the first time, that mitochondrial function may be altered by both tumour burden and immune modulation, follow-up studies along the lines suggested by the reviewer are appropriate.

So, the initial and crucial step for the study is weak. The results obtained from that point pay the price of this initial design. Results on mitochondria function or content are weak and is difficult to conclude if the treatment with anti-PD1 has a crosstalk with exercise effect or not. Moreover, the title Effect of Immune Modulation on the Skeletal Muscle Mitochondrial Exercise Response, appears disconnected with the study, and too much ambitious.

**Our first step was to analyse and confirm adaptation of mitochondrial function in response to the chosen exercise regime in tumour-free mice (Figure 2, added lines 307-309).** We used that basic information to investigate the impact of tumour burden on response to exercise and to design our exploratory study on how immune modulators influenced mitochondrial function in this model system. This has never been attempted before.

As an exploratory study, we agree that our results are not conclusive, but rather hypothesis-generating. This has been clearly stated throughout the manuscript (title, lines 31, 107-109, 563-567, 575-578, 580-582). If our initial findings are confirmed it would have implications for understanding the development of cachexia and the role of exercise in preventing cachexia. This has never been investigated in either animals or humans, and our results suggest that this is a worthy avenue of future research.

Similarly, we are unsure as to why our title appears disconnected with the study and too ambitious. We have not made any claims in the title as to how immunotherapy might affect the exercise response, and we have included the phrase “an exploratory study in mice with cancer” to emphasise that our results are preliminary.

In conclusion, is an important study, that lacks a proper design and execution to answer the question proposed.

We agree that our exploratory study does not conclusively answer the question of whether immunotherapy and cancer alter the skeletal muscle response to exercise, and that it should be followed up with more comprehensive investigations. **This limitation has been clearly stated in lines 563-578.** However, we believe that our work has merit as hypothesis-generating data for other researchers to build on, as it is the first to investigate whether immunotherapy alters skeletal muscle mitochondria.

Additionally, cachexia seems to lose importance along the manuscript. Indeed, Figure 1 indicates that exercise and anti-PD-1, do not alter growth rate of B16-F10 or EO771 tumors. So, we are somewhat lost in the parameter important to be evaluated.

An accelerated tumour growth rate is not necessarily concurrent with the development of cachexia. For example, it has been shown that exercise can delay the onset of cachexia, while not altering tumour incidence (Vanderveen 2020). Therefore, it is valid to investigate changes in cachectic indicators even in the absence of a change in tumour growth rate. **We have added lines 259-260 to clarify this.** In addition, our mice were largely pre-cachectic (line 272), which is why cachexia is not a large focus of the manuscript.

The authors recognize the limitation of the present study, mentioning some of the points expressed above. In my opinion, some of the experiments suggested in their Discussion must be part of the present manuscript.

It appears to us that the main limitation for this reviewer was a weak initial step; we hope we have been able to convince the reviewer that this initial step has in fact been carried out in the form of analysis of mitochondrial function in tumour-free, untreated mice (Figure 2). The suggested experiments are clearly beyond the scope of a major revision of this manuscript as they would require a significant additional time and funding commitment.

One of the conclusions of this study is that “in mice with B16-F10 tumors, IgG2a administration prevents exercise adaptation of skeletal muscle mitochondria, but adaptation remains intact in mice receiving anti-PD-1”. This is clearly one interpretation of the data but is not the only one. The connection of this conclusion with this other: “In mice with EO771 tumors, both IgG2a and anti-PD-1-treated mice show robust skeletal muscle mitochondrial exercise responses, while untreated mice do not” is not present in the manuscript. There is a difference between different tumor types? What is the possible hypothesis for that? What are the control experiments that must be done for probe these hypothesis?

This is a very interesting point, and **we have added a paragraph discussing one possible hypothesis as to why IgG2a has different effects on mice with B16-F10 compared with EO771 tumors (lines 550-562)**.

We have made every effort to follow the reviewers’ suggestions and hope that we have amended the manuscript to the reviewers’ satisfaction. Please do not hesitate to contact me if there are any further questions.

Yours sincerely,

Dr Linda Buss
